# Supplementary material for: Antibiotic exposure enriches streptococci carrying resistance genes in periodontitis plaque biofilms
Source: PeerJ. 2025 Jan 20;13:e18835. doi: 10.7717/peerj.18835 (PMC11756365; doi:10.7717/peerj.18835)
Supplement: Supplemental Information 1 [file peerj-13-18835-s001.docx]

**SI Materials and Methods**

**16S rRNA gene amplicon sequencing**

Bacteria were harvested by centrifugation (8000 rpm, 10 min), followed by DNA extraction using a QIAamp DNA Mini Kit (Qiagen, Hilden, Germany). DNA quality was checked by agarose gel electrophoresis and the NanoDrop 8000 spectrophotometer (Thermo Fisher Scientific). High-quality DNA (OD_260_/OD_280_ =1.8–2.0) with concentration > 20 ng/μL were used for 16S rRNA gene V3-V4 region amplification. The primers were 341F (5'-CCTACGGGRSGCAGCAG-3') and 806R (5'‑GGACTACVVGGGTATCTAATC‑3'), in which barcodes were added to the 5' ends to distinguish the samples. The polymerase chain reaction (PCR) conditions were 95°C for 10 min; 25 cycles of denaturation at 95°C for 30 s, annealing at 55°C for 30 s, and extension at 72°C for 30s; and a final extension at 72°C for 10 min. The products were purified using the AxyPrepDNA Gel Extraction Kit (Axygen Biosciences, Inc., California, USA) and pooled to create libraries with the same proportions in accordance with the manufacturer’s instructions. All the extraction products were identified by Qubit Fluorometer (Invitrogen, USA). The libraries were sequenced using a paired-end 250 bp pattern on the Illumina Miseq platform at Shanghai Biozeron Biotechnology Co., Ltd. (Shanghai, China).

**Whole genome sequencing**

DNA samples were fragmented into 350-450 bp using a Bioruptor sonicator (Diagenode), and libraries were prepared using a NEXTflex Rapid DNA-Seq Kit (Bioo Scientific, Austin, TX) following the manufacturer’s instructions. The quantity of each library was evaluated by Qubit fluorometer (Thermo Fisher Scientific) and sequenced on the Illumina HiSeq X-ten platform using a paired-end 150 bp pattern at Microbial Genome Research Centre, Institute of Microbiology, Chinese Academy of Sciences.

***In vitro* evaluation of resistance phenotypes**

2.5 mL antibiotic solution was mixed with 22.5 mL Mueller–Hinton agar to create test plates with 1-128 μg/mL antibiotic. The strains were activated twice in BHI broth and adjusted to OD_630_ ≈ 0.06 (~1 × 10^8^ colony-forming units/mL). Next, 5 μL bacterial solution was inoculated on each test plate, and a plate without tetracycline was used as the control. After 18-20 h of incubation at 37°C in a CO_2_ incubator, the lowest tetracycline concentration that resulted in fewer than 30 colonies were defined as the minimum inhibitory concentration (MIC). All experiments were performed in triplicate.

**Sequencing data analysis**

16S rRNA gene amplicon sequencing data were processed using QIIME v1.9.1 ^1^. In brief, raw reads were filtered and spliced based on a quality score > 20 and overlap > 10 bp, with no mismatches on barcodes and less than two mismatches between primers. The merged sequences were clustered into operational taxonomic units (OTUs) with 97% similarity. Chimeras and singletons were removed by OTU cluster analysis. OTUs were assigned to taxa via basic local alignment search tool (BLAST) search of the ribosomal database project (RDP) reference database ^2^ and classified into phylum to genus levels. The numbers of sequences were normalized to 100,000. And then, alpha diversity of each sample and Bray-Curtis distances between samples were calculated using the R package Vegan. The comparison of alpha diversity and distance was conducted by t-test. Principal coordinate analysis (PCoA) was used to compare community structures among the groups, with the significance was determined by the nonparametric Adonis test. LEfSe was used to identify the enriched or depleted bacteria between groups. Linear discriminant analysis (LDA) score > 2.0 were considered as a discriminatory signature between different antibiotic treatments.

Metagenomic raw reads were trimmed using Sickle (<https://github.com/najoshi/sickle/>) with the default parameters, and their quality was evaluated using FastQC 0.11.3 (<http://www.bioinformatics.babraham.ac.uk/projects/fastqc/>). All processed reads were assembled into contigs using MEGAHIT ^3^ with the default parameters. Taxator-tk was used to perform taxonomic profiling of contigs ^4^. All genes were predicted from assembled contigs using Prodigal ^5^. And then, matched genes were identified under the criterion of query coverage > 60% and identity > 40% after blasting against the comprehensive antibiotic resistance database (CARD) 3.0.029 using DIAMOND ^6^. High-quality reads were mapped to these genes using Bowtie v10.3.0 software ^7^ and the read coverage of ARGs was evaluated using SAMtools ^8^. Potential functional domains in these genes were identified using the Pfam database (<http://pfam.xfam.org/>) ^9^.

For further *tet*M distribution analysis, all coding sequences from the HMASM of human microbiome project (HMP) database (https://www.hmpdacc.org/HMASM/#data) first were downloaded and blasted against *tet*M gene to screen out the target sequences, taking 95% sequence identity and 70% coverage as the threshold. Then all the target sequences were integrated by using the faSomeRecords script, and the phylogenetic tree of *tet*M gene was built using Mega7 software ^10^. To show the species affiliation and body site distribution of each gene, iTOL (https://itol.embl.de/) and Rstudio tools were used. Also, the detection rate of *tet*M genes from different body sites were calculated based on the target sequences from the HMASM database.

Totally 305 metagenomic sequences related to periodontitis and healthy were retrieved from NCBI and the related clinical information was obtained (Table S1). The downloaded sequences were mapped to *tet*M genes with Bowtie v10.3.0 software ^7^. To eliminate the influence of file size on the number of mapping, normalization was carried out with the number of reads mapping to *tet*M genes counted per every 10^7^ reads using Bowtie v10.3.0 software ^7^.

**Reference**

1. Caporaso JG, Kuczynski J, Stombaugh J, et al. QIIME allows analysis of high-throughput community sequencing data. *Nature methods* 2010;7:335-336.

2. Cole JR, Wang Q, Fish JA, et al. Ribosomal Database Project: data and tools for high throughput rRNA analysis. *Nucleic acids research* 2014;42:D633-642.

3. Li D, Liu CM, Luo R, Sadakane K, Lam TW. MEGAHIT: an ultra-fast single-node solution for large and complex metagenomics assembly via succinct de Bruijn graph. *Bioinformatics (Oxford, England)* 2015;31:1674-1676.

4. Dröge J, Gregor I, McHardy AC. Taxator-tk: precise taxonomic assignment of metagenomes by fast approximation of evolutionary neighborhoods. *Bioinformatics (Oxford, England)* 2015;31:817-824.

5. Hyatt D, Chen GL, Locascio PF, Land ML, Larimer FW, Hauser LJ. Prodigal: prokaryotic gene recognition and translation initiation site identification. *BMC bioinformatics* 2010;11:119.

6. Buchfink B, Xie C, Huson DH. Fast and sensitive protein alignment using DIAMOND. *Nature methods* 2015;12:59-60.

7. Langmead B, Salzberg SL. Fast gapped-read alignment with Bowtie 2. *Nature methods* 2012;9:357-359.

8. Li H, Handsaker B, Wysoker A, et al. The Sequence Alignment/Map format and SAMtools. *Bioinformatics (Oxford, England)* 2009;25:2078-2079.

9. Mistry J, Chuguransky S, Williams L, et al. Pfam: The protein families database in 2021. *Nucleic acids research* 2021;49:D412-d419.

10. Kumar S, Stecher G, Tamura K. MEGA7: Molecular Evolutionary Genetics Analysis Version 7.0 for Bigger Datasets. *Molecular biology and evolution* 2016;33:1870-1874.

**SI Tables**

**Table S1 Full-length and real-time (RT) quantitative polymerase chain reaction primers sequences of *tet*M and 16S rRNA**

| **Genes** | **RT qPCR primers sequences** | **Full-length primers sequences** |
| --- | --- | --- |
| ***tet*M** | **F**: GTGCGAACATCGTAGACACTCAA  **R**: ATCTATCCGACTATTTGGACGAC | **F**: ATGAAAATTATTAATATAGGTGTTTTAGC  **R**: CTAAGTTATTTTATTGAACATATATCGTAC |
| **16S rRNA** | **F**: CCTACGGGAGGCAGCAGTAG  **R**: CAACAGAGCTTTACGATCCGAAA |  |

**Table S2 Metagenomic sample list from public database**

| **BioProject** | **SRA accession** | **health status** | **Sampling site** | **Reads count(#)** | |
| --- | --- | --- | --- | --- | --- |
| PRJNA741688 | SRR15001307 | healthy | saliva | 83193868 |  |
| PRJNA741688 | SRR15001308 | healthy | saliva | 77569396 |  |
| PRJNA741688 | SRR15001309 | healthy | saliva | 75598312 |  |
| PRJNA741688 | SRR15001310 | healthy | saliva | 77508130 |  |
| PRJNA741688 | SRR15001319 | healthy | saliva | 80569578 |  |
| PRJNA741688 | SRR15001320 | healthy | saliva | 81820866 |  |
| PRJNA230363 | SRR2037087 | healthy | saliva | 35732226 |  |
| PRJNA230363 | SRR2037088 | healthy | saliva | 94503552 |  |
| PRJNA230363 | SRR2037089 | healthy | saliva | 222827944 |  |
| PRJNA230363 | SRR2037090 | healthy | saliva | 229910704 |  |
| PRJNA230363 | SRR2037091 | healthy | saliva | 39440516 |  |
| PRJNA741688 | SRR15001303 | periodontitis | saliva | 78025316 |  |
| PRJNA741688 | SRR15001304 | periodontitis | saliva | 75790690 |  |
| PRJNA741688 | SRR15001305 | periodontitis | saliva | 119194488 |  |
| PRJNA741688 | SRR15001306 | periodontitis | saliva | 79368758 |  |
| PRJNA741688 | SRR15001311 | periodontitis | saliva | 87792368 |  |
| PRJNA741688 | SRR15001312 | periodontitis | saliva | 83083908 |  |
| PRJNA741688 | SRR15001313 | periodontitis | saliva | 74260562 |  |
| PRJNA741688 | SRR15001314 | periodontitis | saliva | 76629884 |  |
| PRJNA741688 | SRR15001315 | periodontitis | saliva | 78788086 |  |
| PRJNA741688 | SRR15001316 | periodontitis | saliva | 78669706 |  |
| PRJNA741688 | SRR15001317 | periodontitis | saliva | 74618136 |  |
| PRJNA741688 | SRR15001318 | periodontitis | saliva | 77793184 |  |
| PRJNA396840 | SRR5892193 | periodontitis | saliva | 10647294 |  |
| PRJNA396840 | SRR5892194 | periodontitis | saliva | 6258030 |  |
| PRJNA396840 | SRR5892196 | periodontitis | saliva | 7787328 |  |
| PRJNA396840 | SRR5892197 | periodontitis | saliva | 2940576 |  |
| PRJNA396840 | SRR5892198 | periodontitis | saliva | 9813916 |  |
| PRJNA396840 | SRR5892199 | periodontitis | saliva | 4899960 |  |
| PRJNA396840 | SRR5892201 | periodontitis | saliva | 4882210 |  |
| PRJNA396840 | SRR5892202 | periodontitis | saliva | 4950934 |  |
| PRJNA396840 | SRR5892203 | periodontitis | saliva | 3130690 |  |
| PRJNA396840 | SRR5892206 | periodontitis | saliva | 3657842 |  |
| PRJNA396840 | SRR5892208 | periodontitis | saliva | 3370786 |  |
| PRJNA396840 | SRR5892209 | periodontitis | saliva | 8975816 |  |
| PRJNA396840 | SRR5892210 | periodontitis | saliva | 12037902 |  |
| PRJNA396840 | SRR5892211 | periodontitis | saliva | 7759494 |  |
| PRJNA396840 | SRR5892212 | periodontitis | saliva | 3323092 |  |
| PRJNA396840 | SRR5892213 | periodontitis | saliva | 8550254 |  |
| PRJNA396840 | SRR5892214 | periodontitis | saliva | 9257802 |  |
| PRJNA396840 | SRR5892215 | periodontitis | saliva | 12425918 |  |
| PRJNA396840 | SRR5892216 | periodontitis | saliva | 14434618 |  |
| PRJNA396840 | SRR5892217 | periodontitis | saliva | 6096682 |  |
| PRJEB42701 | ERR5383528 | periodontitis | saliva | 40823338 |  |
| PRJEB42701 | ERR5383531 | periodontitis | saliva | 46723008 |  |
| PRJEB42701 | ERR5383532 | periodontitis | saliva | 30104422 |  |
| PRJEB42701 | ERR5383534 | periodontitis | saliva | 33303580 |  |
| PRJEB42701 | ERR5384476 | periodontitis | saliva | 36375268 |  |
| PRJEB42701 | ERR5384527 | periodontitis | saliva | 46979588 |  |
| PRJEB42701 | ERR5384690 | periodontitis | saliva | 37901636 |  |
| PRJEB42701 | ERR5385017 | periodontitis | saliva | 35594796 |  |
| PRJEB42701 | ERR5385035 | periodontitis | saliva | 34956926 |  |
| PRJEB42701 | ERR5385042 | periodontitis | saliva | 36554766 |  |
| PRJEB42701 | ERR5385085 | periodontitis | saliva | 32485308 |  |
| PRJEB42701 | ERR5385087 | periodontitis | saliva | 38321614 |  |
| PRJEB42701 | ERR5385088 | periodontitis | saliva | 57641882 |  |
| PRJEB42701 | ERR5385092 | periodontitis | saliva | 39159620 |  |
| PRJEB42701 | ERR5385093 | periodontitis | saliva | 42389118 |  |
| PRJNA78025 | SRR653444 | healthy | plaque | 12504058 |  |
| PRJNA78025 | SRR653445 | healthy | plaque | 1130070 |  |
| PRJNA78025 | SRR653447 | healthy | plaque | 3683652 |  |
| PRJNA78025 | SRR653448 | healthy | plaque | 4593298 |  |
| PRJNA78025 | SRR653451 | healthy | plaque | 487310 |  |
| PRJNA78025 | SRR653452 | healthy | plaque | 501442 |  |
| PRJNA528558 | SRR8786264 | healthy | plaque | 30834978 |  |
| PRJNA528558 | SRR8786265 | healthy | plaque | 30407662 |  |
| PRJNA528558 | SRR8786267 | healthy | plaque | 30748896 |  |
| PRJNA508385 | SRR9641788 | healthy | plaque | 1831606 |  |
| PRJNA508385 | SRR9641789 | healthy | plaque | 4376658 |  |
| PRJNA508385 | SRR9641790 | healthy | plaque | 2881482 |  |
| PRJNA508385 | SRR9641791 | healthy | plaque | 2600836 |  |
| PRJNA508385 | SRR9641792 | healthy | plaque | 2745754 |  |
| PRJNA508385 | SRR9641793 | healthy | plaque | 3675050 |  |
| PRJNA508385 | SRR9641794 | healthy | plaque | 2860504 |  |
| PRJNA508385 | SRR9641795 | healthy | plaque | 3004178 |  |
| PRJNA508385 | SRR9641796 | healthy | plaque | 3560200 |  |
| PRJNA508385 | SRR9641797 | healthy | plaque | 2334364 |  |
| PRJNA508385 | SRR9641798 | healthy | plaque | 2367130 |  |
| PRJNA508385 | SRR9641799 | healthy | plaque | 3216546 |  |
| PRJNA508385 | SRR9641800 | healthy | plaque | 3073714 |  |
| PRJNA508385 | SRR9641801 | healthy | plaque | 2599898 |  |
| PRJNA508385 | SRR9641802 | healthy | plaque | 2650630 |  |
| PRJNA508385 | SRR9641803 | healthy | plaque | 2579390 |  |
| PRJNA508385 | SRR9641804 | healthy | plaque | 3322750 |  |
| PRJNA508385 | SRR9641805 | healthy | plaque | 1941224 |  |
| PRJNA508385 | SRR9641806 | healthy | plaque | 3730134 |  |
| PRJNA508385 | SRR9641807 | healthy | plaque | 2195438 |  |
| PRJNA508385 | SRR9641808 | healthy | plaque | 1962534 |  |
| PRJNA508385 | SRR9641809 | healthy | plaque | 2987446 |  |
| PRJNA508385 | SRR9641810 | healthy | plaque | 4715914 |  |
| PRJNA508385 | SRR9641811 | healthy | plaque | 3414446 |  |
| PRJNA508385 | SRR9641812 | healthy | plaque | 3917774 |  |
| PRJNA255922 | SRR10276558 | healthy | plaque | 26003180 |  |
| PRJNA255922 | SRR10276559 | healthy | plaque | 23854744 |  |
| PRJNA255922 | SRR10276560 | healthy | plaque | 9266032 |  |
| PRJNA255922 | SRR10276561 | healthy | plaque | 26827034 |  |
| PRJNA255922 | SRR10276562 | healthy | plaque | 8026930 |  |
| PRJNA255922 | SRR10276563 | healthy | plaque | 17083308 |  |
| PRJNA255922 | SRR10276564 | healthy | plaque | 6676474 |  |
| PRJNA255922 | SRR10276565 | healthy | plaque | 1801032 |  |
| PRJNA255922 | SRR10276566 | healthy | plaque | 31749888 |  |
| PRJNA255922 | SRR10276567 | healthy | plaque | 6135160 |  |
| PRJNA255922 | SRR10276568 | healthy | plaque | 2388296 |  |
| PRJNA255922 | SRR10276569 | healthy | plaque | 26541204 |  |
| PRJNA255922 | SRR10276570 | healthy | plaque | 25122704 |  |
| PRJNA255922 | SRR10276571 | healthy | plaque | 15939814 |  |
| PRJNA255922 | SRR10276572 | healthy | plaque | 27687256 |  |
| PRJNA255922 | SRR10276573 | healthy | plaque | 10500958 |  |
| PRJNA255922 | SRR10276574 | healthy | plaque | 17277636 |  |
| PRJNA255922 | SRR10276575 | healthy | plaque | 25871498 |  |
| PRJNA255922 | SRR10276576 | healthy | plaque | 27283762 |  |
| PRJNA255922 | SRR10276577 | healthy | plaque | 9801998 |  |
| PRJNA255922 | SRR10276578 | healthy | plaque | 10662198 |  |
| PRJNA255922 | SRR10276579 | healthy | plaque | 37872660 |  |
| PRJNA255922 | SRR10276580 | healthy | plaque | 7517036 |  |
| PRJNA255922 | SRR10276581 | healthy | plaque | 24342880 |  |
| PRJNA255922 | SRR10276582 | healthy | plaque | 9948702 |  |
| PRJNA255922 | SRR10276583 | healthy | plaque | 17561572 |  |
| PRJNA255922 | SRR10276584 | healthy | plaque | 14433704 |  |
| PRJNA255922 | SRR10276585 | healthy | plaque | 7650468 |  |
| PRJNA255922 | SRR10276586 | healthy | plaque | 38461650 |  |
| PRJNA255922 | SRR10276589 | healthy | plaque | 25285772 |  |
| PRJNA255922 | SRR10276590 | healthy | plaque | 21320148 |  |
| PRJNA255922 | SRR10276591 | healthy | plaque | 8271732 |  |
| PRJNA255922 | SRR10276592 | healthy | plaque | 22964524 |  |
| PRJNA255922 | SRR10276593 | healthy | plaque | 18019732 |  |
| PRJNA255922 | SRR10276594 | healthy | plaque | 9526194 |  |
| PRJNA255922 | SRR10276595 | healthy | plaque | 21843574 |  |
| PRJNA255922 | SRR10276596 | healthy | plaque | 35103376 |  |
| PRJNA255922 | SRR10276597 | healthy | plaque | 7632868 |  |
| PRJNA255922 | SRR10276598 | healthy | plaque | 28381010 |  |
| PRJNA255922 | SRR10276599 | healthy | plaque | 25591830 |  |
| PRJNA255922 | SRR10276600 | healthy | plaque | 18244242 |  |
| PRJNA255922 | SRR10276601 | healthy | plaque | 43171906 |  |
| PRJNA255922 | SRR10276602 | healthy | plaque | 44031606 |  |
| PRJNA255922 | SRR10276603 | healthy | plaque | 27288286 |  |
| PRJNA255922 | SRR10276604 | healthy | plaque | 26906164 |  |
| PRJNA230363 | SRR1045092 | healthy | plaque | 9792960 |  |
| PRJNA230363 | SRR1045093 | healthy | plaque | 10574606 |  |
| PRJNA230363 | SRR1045094 | healthy | plaque | 8892556 |  |
| PRJNA230363 | SRR1045095 | healthy | plaque | 8122434 |  |
| PRJNA230363 | SRR1045096 | healthy | plaque | 11931698 |  |
| PRJNA230363 | SRR1045097 | healthy | plaque | 15200260 |  |
| PRJNA230363 | SRR2034640 | healthy | plaque | 15275976 |  |
| PRJNA78025 | SRR651965 | periodontitis | plaque | 7258054 |  |
| PRJNA78025 | SRR653183 | periodontitis | plaque | 8377084 |  |
| PRJNA78025 | SRR653184 | periodontitis | plaque | 1081120 |  |
| PRJNA78025 | SRR653185 | periodontitis | plaque | 1194120 |  |
| PRJNA78025 | SRR653413 | periodontitis | plaque | 6674292 |  |
| PRJNA78025 | SRR653414 | periodontitis | plaque | 7435332 |  |
| PRJNA78025 | SRR653417 | periodontitis | plaque | 957254 |  |
| PRJNA78025 | SRR653418 | periodontitis | plaque | 697236 |  |
| PRJNA552294 | SRR9684942 | periodontitis | plaque | 17511628 |  |
| PRJNA552294 | SRR9684943 | periodontitis | plaque | 18511342 |  |
| PRJNA552294 | SRR9684944 | periodontitis | plaque | 16759526 |  |
| PRJNA552294 | SRR9684945 | periodontitis | plaque | 17315650 |  |
| PRJNA552294 | SRR9684946 | periodontitis | plaque | 13185154 |  |
| PRJNA552294 | SRR9684947 | periodontitis | plaque | 16183864 |  |
| PRJNA552294 | SRR9684948 | periodontitis | plaque | 11003934 |  |
| PRJNA552294 | SRR9684949 | periodontitis | plaque | 17521580 |  |
| PRJNA552294 | SRR9684950 | periodontitis | plaque | 11773378 |  |
| PRJNA552294 | SRR9684951 | periodontitis | plaque | 17786822 |  |
| PRJNA552294 | SRR9684952 | periodontitis | plaque | 13352218 |  |
| PRJNA552294 | SRR9684953 | periodontitis | plaque | 13453786 |  |
| PRJNA552294 | SRR9684954 | periodontitis | plaque | 12262904 |  |
| PRJNA552294 | SRR9684955 | periodontitis | plaque | 12279870 |  |
| PRJNA552294 | SRR9684956 | periodontitis | plaque | 12772938 |  |
| PRJNA552294 | SRR9684957 | periodontitis | plaque | 10797762 |  |
| PRJNA552294 | SRR9684958 | periodontitis | plaque | 13572596 |  |
| PRJNA552294 | SRR9684959 | periodontitis | plaque | 11128322 |  |
| PRJNA552294 | SRR9684960 | periodontitis | plaque | 11982688 |  |
| PRJNA552294 | SRR9684961 | periodontitis | plaque | 12529208 |  |
| PRJNA552294 | SRR9684962 | periodontitis | plaque | 13647746 |  |
| PRJNA552294 | SRR9684963 | periodontitis | plaque | 16816618 |  |
| PRJNA552294 | SRR9684964 | periodontitis | plaque | 13282698 |  |
| PRJNA552294 | SRR9684965 | periodontitis | plaque | 18947852 |  |
| PRJNA552294 | SRR9684966 | periodontitis | plaque | 11605262 |  |
| PRJNA552294 | SRR9684967 | periodontitis | plaque | 12532028 |  |
| PRJNA552294 | SRR9684968 | periodontitis | plaque | 18163292 |  |
| PRJNA552294 | SRR9684969 | periodontitis | plaque | 12700702 |  |
| PRJNA552294 | SRR9684970 | periodontitis | plaque | 10514016 |  |
| PRJNA552294 | SRR9684971 | periodontitis | plaque | 15892370 |  |
| PRJNA552294 | SRR9684972 | periodontitis | plaque | 11497702 |  |
| PRJNA552294 | SRR9684973 | periodontitis | plaque | 21599716 |  |
| PRJNA552294 | SRR9684974 | periodontitis | plaque | 17883006 |  |
| PRJNA552294 | SRR9684975 | periodontitis | plaque | 22516062 |  |
| PRJNA552294 | SRR9684976 | periodontitis | plaque | 20618842 |  |
| PRJNA552294 | SRR9684977 | periodontitis | plaque | 16898722 |  |
| PRJNA552294 | SRR9684978 | periodontitis | plaque | 17035472 |  |
| PRJNA552294 | SRR9684979 | periodontitis | plaque | 19128266 |  |
| PRJNA552294 | SRR9684980 | periodontitis | plaque | 25664146 |  |
| PRJNA552294 | SRR9684981 | periodontitis | plaque | 7152130 |  |
| PRJNA552294 | SRR9684982 | periodontitis | plaque | 20701570 |  |
| PRJNA552294 | SRR9684983 | periodontitis | plaque | 18423716 |  |
| PRJNA552294 | SRR9684984 | periodontitis | plaque | 13518918 |  |
| PRJNA552294 | SRR9684985 | periodontitis | plaque | 12368996 |  |
| PRJNA552294 | SRR9684986 | periodontitis | plaque | 12882342 |  |
| PRJNA552294 | SRR9684987 | periodontitis | plaque | 15799976 |  |
| PRJNA552294 | SRR9684988 | periodontitis | plaque | 18095692 |  |
| PRJNA552294 | SRR9684989 | periodontitis | plaque | 11052680 |  |
| PRJNA552294 | SRR9684990 | periodontitis | plaque | 10891084 |  |
| PRJNA552294 | SRR9684991 | periodontitis | plaque | 10554602 |  |
| PRJNA552294 | SRR9684992 | periodontitis | plaque | 12095734 |  |
| PRJNA552294 | SRR9684993 | periodontitis | plaque | 13478846 |  |
| PRJNA552294 | SRR9684994 | periodontitis | plaque | 21662242 |  |
| PRJNA552294 | SRR9684995 | periodontitis | plaque | 16663284 |  |
| PRJNA552294 | SRR9684996 | periodontitis | plaque | 13013866 |  |
| PRJNA552294 | SRR9684997 | periodontitis | plaque | 20423422 |  |
| PRJNA552294 | SRR9684998 | periodontitis | plaque | 17329788 |  |
| PRJNA552294 | SRR9684999 | periodontitis | plaque | 20454882 |  |
| PRJNA552294 | SRR9685000 | periodontitis | plaque | 12496652 |  |
| PRJNA552294 | SRR9685001 | periodontitis | plaque | 12141426 |  |
| PRJNA552294 | SRR9685002 | periodontitis | plaque | 12356982 |  |
| PRJNA552294 | SRR9685003 | periodontitis | plaque | 11724518 |  |
| PRJNA552294 | SRR9685004 | periodontitis | plaque | 12684294 |  |
| PRJNA552294 | SRR9685005 | periodontitis | plaque | 12492568 |  |
| PRJNA552294 | SRR9685006 | periodontitis | plaque | 12142766 |  |
| PRJNA552294 | SRR9685007 | periodontitis | plaque | 16370804 |  |
| PRJNA552294 | SRR9685008 | periodontitis | plaque | 13402952 |  |
| PRJNA552294 | SRR9685009 | periodontitis | plaque | 13565222 |  |
| PRJNA528558 | SRR8786260 | periodontitis | plaque | 30825240 |  |
| PRJNA528558 | SRR8786261 | periodontitis | plaque | 30912934 |  |
| PRJNA528558 | SRR8786262 | periodontitis | plaque | 30633470 |  |
| PRJNA528558 | SRR8786263 | periodontitis | plaque | 29988486 |  |
| PRJNA528558 | SRR8786266 | periodontitis | plaque | 28753246 |  |
| PRJNA528558 | SRR8786268 | periodontitis | plaque | 29782530 |  |
| PRJNA255922 | SRR1779378 | periodontitis | plaque | 47677480 |  |
| PRJNA255922 | SRR1779403 | periodontitis | plaque | 68198196 |  |
| PRJNA255922 | SRR1779468 | periodontitis | plaque | 12639030 |  |
| PRJNA255922 | SRR1779479 | periodontitis | plaque | 17622330 |  |
| PRJNA255922 | SRR1779482 | periodontitis | plaque | 13771922 |  |
| PRJNA255922 | SRR1779488 | periodontitis | plaque | 40492790 |  |
| PRJNA255922 | SRR1779492 | periodontitis | plaque | 22751752 |  |
| PRJNA255922 | SRR1779495 | periodontitis | plaque | 7039766 |  |
| PRJNA255922 | SRR1779497 | periodontitis | plaque | 50132572 |  |
| PRJNA255922 | SRR1779502 | periodontitis | plaque | 56218562 |  |
| PRJNA255922 | SRR1779506 | periodontitis | plaque | 25668576 |  |
| PRJNA255922 | SRR1779511 | periodontitis | plaque | 6939962 |  |
| PRJNA255922 | SRR1781914 | periodontitis | plaque | 57873198 |  |
| PRJNA255922 | SRR1781969 | periodontitis | plaque | 39124116 |  |
| PRJNA255922 | SRR1781970 | periodontitis | plaque | 9407334 |  |
| PRJNA255922 | SRR1781983 | periodontitis | plaque | 55459404 |  |
| PRJNA255922 | SRR1783028 | periodontitis | plaque | 40639486 |  |
| PRJNA255922 | SRR1783032 | periodontitis | plaque | 22257874 |  |
| PRJNA255922 | SRR1783079 | periodontitis | plaque | 57182684 |  |
| PRJNA255922 | SRR1783718 | periodontitis | plaque | 31813530 |  |
| PRJNA255922 | SRR1783722 | periodontitis | plaque | 17267402 |  |
| PRJNA255922 | SRR1783724 | periodontitis | plaque | 44928046 |  |
| PRJNA255922 | SRR1783757 | periodontitis | plaque | 49870904 |  |
| PRJNA255922 | SRR1783758 | periodontitis | plaque | 72980664 |  |
| PRJNA255922 | SRR1783760 | periodontitis | plaque | 25785142 |  |
| PRJNA255922 | SRR1783763 | periodontitis | plaque | 15215256 |  |
| PRJNA255922 | SRR1783767 | periodontitis | plaque | 67791904 |  |
| PRJNA255922 | SRR1783768 | periodontitis | plaque | 13035620 |  |
| PRJNA255922 | SRR1783769 | periodontitis | plaque | 46150292 |  |
| PRJNA255922 | SRR1783771 | periodontitis | plaque | 22832140 |  |
| PRJNA255922 | SRR1783774 | periodontitis | plaque | 61285758 |  |
| PRJNA255922 | SRR1783775 | periodontitis | plaque | 42480598 |  |
| PRJNA255922 | SRR1794083 | periodontitis | plaque | 92621186 |  |
| PRJNA255922 | SRR1794618 | periodontitis | plaque | 86702108 |  |
| PRJNA255922 | SRR1794621 | periodontitis | plaque | 11658042 |  |
| PRJNA255922 | SRR1794623 | periodontitis | plaque | 32965038 |  |
| PRJNA255922 | SRR1794794 | periodontitis | plaque | 18818890 |  |
| PRJNA255922 | SRR1795007 | periodontitis | plaque | 64819738 |  |
| PRJNA255922 | SRR1795008 | periodontitis | plaque | 56106866 |  |
| PRJNA255922 | SRR1795009 | periodontitis | plaque | 66035244 |  |
| PRJNA255922 | SRR1795012 | periodontitis | plaque | 15578194 |  |
| PRJNA255922 | SRR1795016 | periodontitis | plaque | 31778656 |  |
| PRJNA255922 | SRR1795017 | periodontitis | plaque | 22208152 |  |
| PRJNA255922 | SRR1795018 | periodontitis | plaque | 69712256 |  |
| PRJNA255922 | SRR1795019 | periodontitis | plaque | 53684366 |  |
| PRJNA255922 | SRR1795020 | periodontitis | plaque | 35702656 |  |
| PRJNA255922 | SRR1795229 | periodontitis | plaque | 61815416 |  |
| PRJNA255922 | SRR1795230 | periodontitis | plaque | 60706608 |  |
| PRJNA230363 | SRR1044006 | periodontitis | plaque | 18519330 |  |
| PRJNA230363 | SRR1044017 | periodontitis | plaque | 7600298 |  |
| PRJNA230363 | SRR1044032 | periodontitis | plaque | 8313926 |  |
| PRJNA230363 | SRR1044033 | periodontitis | plaque | 11841736 |  |
| PRJNA230363 | SRR1044034 | periodontitis | plaque | 13307752 |  |
| PRJNA230363 | SRR2034637 | periodontitis | plaque | 8073004 |  |
| PRJNA230363 | SRR2034638 | periodontitis | plaque | 11569812 |  |
| PRJNA230363 | SRR2034639 | periodontitis | plaque | 18265188 |  |
| PRJEB42701 | ERR5383527 | periodontitis | plaque | 34757522 |  |
| PRJEB42701 | ERR5383529 | periodontitis | plaque | 43636626 |  |
| PRJEB42701 | ERR5383530 | periodontitis | plaque | 38503096 |  |
| PRJEB42701 | ERR5383533 | periodontitis | plaque | 32641620 |  |
| PRJEB42701 | ERR5384453 | periodontitis | plaque | 47549678 |  |
| PRJEB42701 | ERR5384454 | periodontitis | plaque | 38478304 |  |
| PRJEB42701 | ERR5384455 | periodontitis | plaque | 39484650 |  |
| PRJEB42701 | ERR5384745 | periodontitis | plaque | 34258268 |  |
| PRJEB42701 | ERR5385013 | periodontitis | plaque | 37053446 |  |
| PRJEB42701 | ERR5385032 | periodontitis | plaque | 34632986 |  |
| PRJEB42701 | ERR5385033 | periodontitis | plaque | 41662314 |  |
| PRJEB42701 | ERR5385034 | periodontitis | plaque | 42403088 |  |
| PRJEB42701 | ERR5385043 | periodontitis | plaque | 40204168 |  |
| PRJEB42701 | ERR5385062 | periodontitis | plaque | 41629322 |  |
| PRJEB42701 | ERR5385081 | periodontitis | plaque | 53357040 |  |
| PRJEB42701 | ERR5385082 | periodontitis | plaque | 37345020 |  |
| PRJEB42701 | ERR5385083 | periodontitis | plaque | 47736100 |  |
| PRJEB42701 | ERR5385084 | periodontitis | plaque | 37330962 |  |
| PRJEB42701 | ERR5385086 | periodontitis | plaque | 34060904 |  |
| PRJEB42701 | ERR5385089 | periodontitis | plaque | 44636382 |  |
| PRJEB42701 | ERR5385090 | periodontitis | plaque | 37329238 |  |
| PRJEB42701 | ERR5385091 | periodontitis | plaque | 33093792 |  |

**Table S3 Samples for 16S rRNA gene amplicon and metagenomic sequencing**

| **Samples** | | **Antibotic-**  **free** | **Amoxicillin** | **Metronidazole** | **Clindamycin** | **Tetracycline** |
| --- | --- | --- | --- | --- | --- | --- |
| P1 | ⊕, + | | ⊕, + | + | + | ⊕, + |
| P2 | + | | + | + | + | + |
| P3 | ⊕, + | | ⊕, + | + | + | ⊕, + |
| P4 | + | | + | + | + | + |
| P5 | + | | + | + | + | + |
| P6 | + | | + | + | + | + |
| P7 | ⊕, + | | ⊕, + | + | + | ⊕, + |
| P8 | ⊕, + | | ⊕, + | + | + | ⊕, + |
| P9 | + | | + | + | + | + |
| P10 | ⊕, + | | ⊕, + | + | + | ⊕, + |
| H1 | + | | × | + | + | × |
| H2 | + | | × | + | + | + |
| H3 | + | | × | + | + | + |
| H4 | + | | × | + | + | × |
| H5 | + | | × | + | + | + |
| H6 | + | | × | + | + | × |
| H7 | + | | × | + | + | × |
| H8 | + | | × | + | + | + |
| H9 | + | | × | + | + | × |
| H10 | + | | × | + | × | + |
| H11 | + | | × | × | × | × |

P: periodontitis group; H: health group; ×: no growth under different treatments; +: samples for 16S rRNA gene amplicon sequencing (totally 85 samples); ⊕: metagenomic sequencing (totally 15 samples);

**Table S4 Abundance and annotations of significant differential contigs**

Please refer to the additional excel file.

**Table S5 Resistant and sensitive *Streptococcus* strains isolated in this study and the presence of *tet*M**

| **Isolates number** | **Sample source** | **Besthits in NCBI** | **Presence of *tet*M** | **Resistant or Sensitive** |
| --- | --- | --- | --- | --- |
| P1S3 | P1, tetracycline-treated | *S. sanginosus* | Yes | R |
| P1m8 | P1, untreated control | *S. sanginosus* | No | S |
| P3S1 | P3, tetracycline-treated | *S. salivarius* | Yes | R |
| P3S2 | P3, tetracycline-treated | *S. oralis sub. tigurinus* | Yes | R |
| P3S3 | P3, tetracycline-treated | *S. oralis sub. dentisani* | Yes | R |
| P3y2 | P3, untreated control | *S. anginosus* | No | S |
| P7S2 | P7, tetracycline-treated | *S. sanginosus* | Yes | R |
| P7S3 | P7, tetracycline-treated | *S. oralis sub. tigurinus* | Yes | R |
| P7S4 | P7, tetracycline-treated | *S. sinensis* | Yes | R |
| P7m8 | P7, untreated control | *S. sanginosus* | No | S |
| P8m6 | P8, untreated control | *S. sanginosus* | Yes | R |
| P8m3 | P8, untreated control | *S. sanginosus* | No | S |
| P10S1 | P10, tetracycline-treated | *S. constellatus* | Yes | R |
| P10S4 | P10, tetracycline-treated | *S. gordonii* | Yes | R |
| H1-2 | H1, untreated control | *S. oralis sub. tigurinus* | No | S |
| H1-3 | H1, untreated control | *S. sanginosus* | No | S |
| H2-1 | H2, tetracycline-treated | *S. intermedius* | Yes | R |
| H2-2 | H2, tetracycline-treated | *S. sanginosus* | Yes | R |
| H4-3 | H4, untreated control | *S. oralis sub. tigurinus* | Yes | R |
| H6-2 | H6, untreated control | *S. salivarius* | No | S |
| H7-3 | H7, untreated control | *S. oralis sub. tigurinus* | Yes | R |
| H8-2 | H8, tetracycline-treated | *S. salivarius* | Yes | R |
| H9-4 | H9, untreated control | *S. sanginosus* | Yes | R |
| H11-2 | H11, untreated control | *S. intermedius* | Yes | S |

P: periodontitis group; H: health group; R: resistant; S: sensitive;

**Table S6 Minimum inhibitory concentration (MIC) of selected strains**

| **Isolates number** | **Besthits in NCBI** | **MIC for tetracycline (μg/ ml)** |
| --- | --- | --- |
| P1S3 | *S. sanginosus* | 32 |
| P7S2 | *S. sanginosus* | 32 |
| P8m6 | *S. sanginosus* | 32 |
| H2-2 | *S. sanginosus* | 32 |
| H9-4 | *S. sanginosus* | 32 |
| P1m8 | *S. sanginosus* | <1 |
| P3y2 | *S. sanginosus* | <1 |
| P7m8 | *S. sanginosus* | <1 |
| H1-3 | *S. sanginosus* | <1 |
| P3S2 | *S. oralis sub. tigurinus* | 16 |
| P7S3 | *S. oralis sub. tigurinus* | 64 |
| P3S3 | *S. oralis sub. dentisani* | 32 |
| H4-3 | *S. oralis sub. tigurinus* | 8 |
| H7-3 | *S. oralis sub. tigurinus* | 16 |
| H1-2 | *S. oralis sub. tigurinus* | <1 |
| P3S1 | *S. salivarius* | 64 |
| H8-2 | *S. salivarius* | 16 |
| P8m3 | *S. salivarius* | <1 |
| H6-2 | *S. salivarius* | <1 |
| H2-1 | *S. intermedius* | 16 |
| H11-2 | *S. intermedius* | <1 |
| P10S4 | 1. *gordonii* | 32 |

**SI Figures**


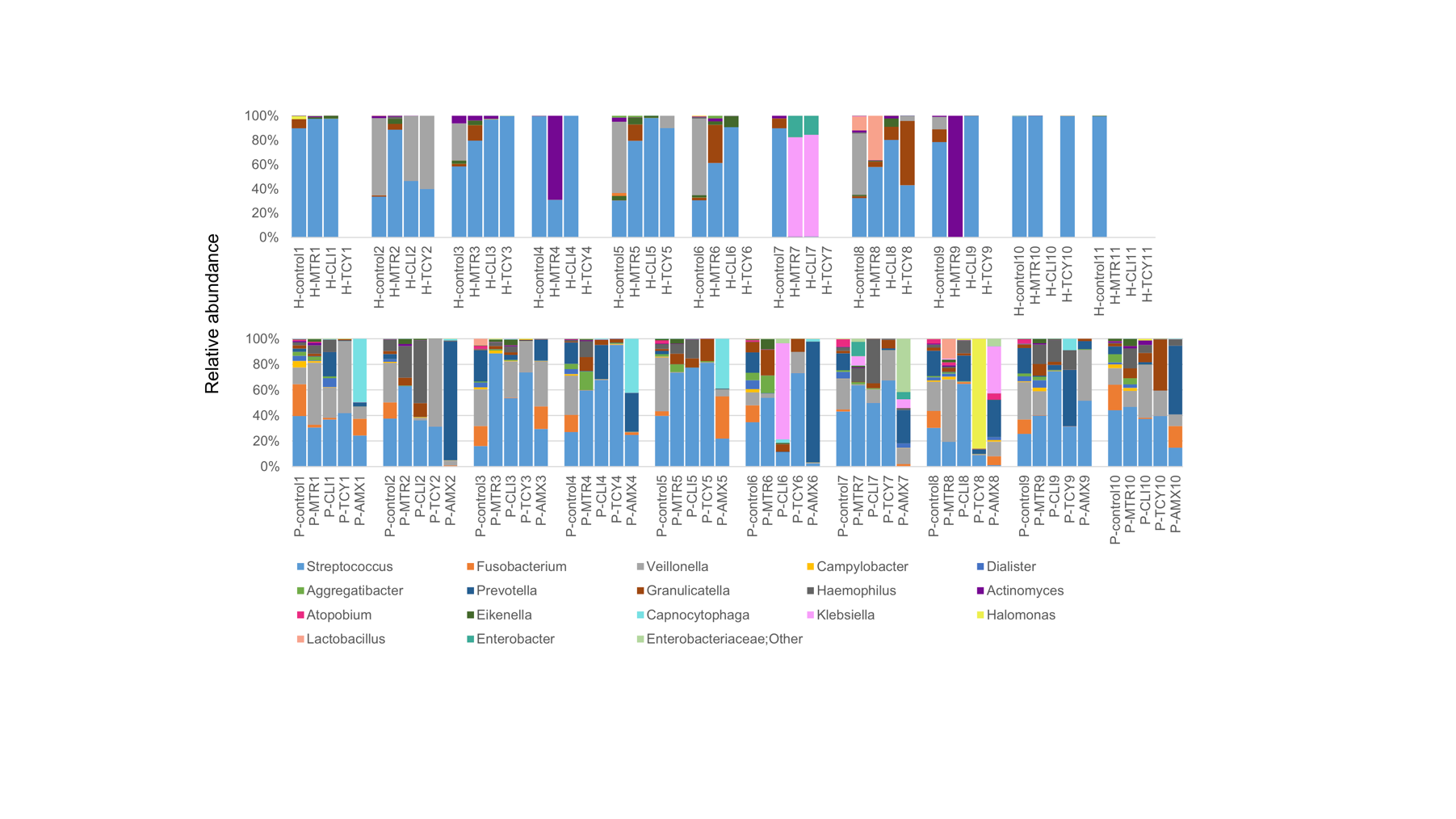


**Fig S1. The taxonomic profiles of each sample at the genus level.**

H: healthy; P: periodontitis; AMX: amoxicillin; MTR: metronidazole; CLI: clindamycin; TCY: tetracycline.


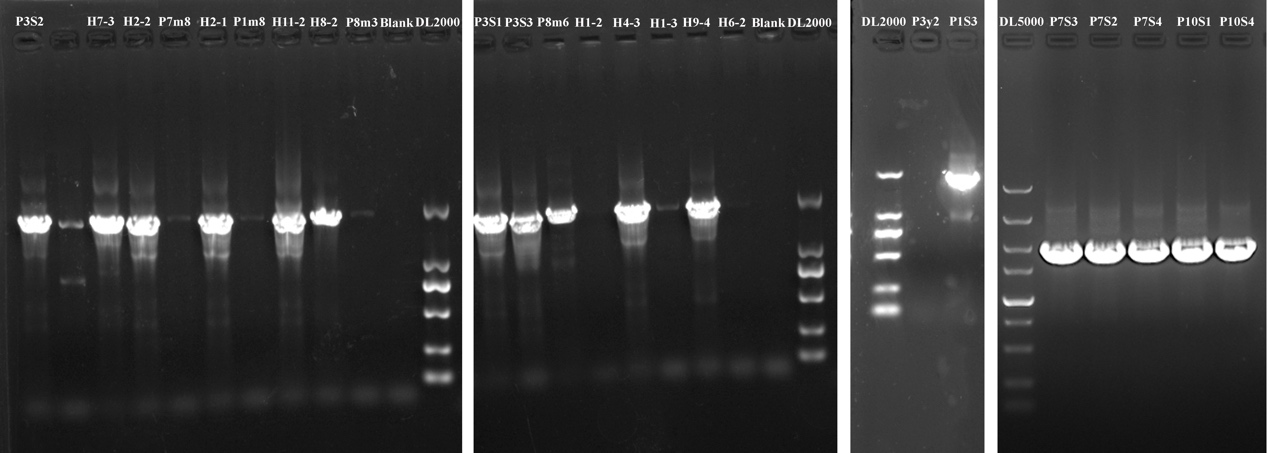


**Fig S2. The gels/blots of *tet*M gene amplification products of isolated bacterial strains.**
