# Supplementary material for: Antibiotic exposure enriches streptococci carrying resistance genes in periodontitis plaque biofilms
Source: PeerJ. 2025 Jan 20;13:e18835. doi: 10.7717/peerj.18835 (PMC11756365; doi:10.7717/peerj.18835)
Supplement: Supplemental Information 3 [file peerj-13-18835-s003.docx]

**Table S4 Abundance and annotations of significant differential contigs**

| **Contig ID** | **CARD term** | **BLAST identity** | **Alignment length** | **Contig length** | **P1-Control** | **P3-Control** | **P7-Control** | **P8-Control** | **P10-Control** | **P1-TCY** | **P3-TCY** | **P7-TCY** | **P8-TCY** | **P10-TCY** | **P1-AMX** | **P3-AMX** | **P7-AMX** | **P8-AMX** | **P10-AMX** | **p-value** | **adjust p-value** | **p-value(TCY vs Control)** | **p-value(AMX vs Control)** |
| --- | --- | --- | --- | --- | --- | --- | --- | --- | --- | --- | --- | --- | --- | --- | --- | --- | --- | --- | --- | --- | --- | --- | --- |
| k141_217576 | gb_AM180355_+_600033-601953_ARO:3000186_tetM | 93.75 | 528 | 528 | 285.9208 | 147.2844 | 211.211 | 64.4437 | 839.1882 | 2836.1777 | 1437.7211 | 1311.4515 | 2409.795 | 612.3211 | 611.4664 | 219.8005 | 43.3569 | 6.8717 | 563.3774 | 0.012778388 | 0.09537497 | 0.01048616 | 0.54251317 |
| k141_330197 | gb_NZ_CP018138.1_-_1692408-1695312_ARO:3004181_Streptococcus | 94.748 | 476 | 618 | 1418.9235 | 706.3967 | 2535.2927 | 1549.2322 | 1432.4425 | 4182.497 | 3261.7558 | 4870.9678 | 3396.0354 | 1109.8025 | 939.5264 | 1280.6524 | 5.5298 | 77.9978 | 1289.8556 | 0.022148179 | 0.098953881 | 0.020744469 | 0.958914868 |
| k141_488546 | gb_AF242872_+_2131-2878_ARO:3000375_ErmB | 99.598 | 747 | 2330 | 758.6943 | 538.6204 | 390.1507 | 263.9515 | 665.5984 | 2966.3381 | 4104.8476 | 3243.6446 | 6131.594 | 803.6372 | 1308.2595 | 739.8058 | 60.897 | 63.4648 | 731.2278 | 0.013167547 | 0.09537497 | 0.01353621 | 0.41460722 |
| k141_615477 | gb_AM180355_+_600033-601953_ARO:3000186_tetM | 97.059 | 408 | 890 | 667.062 | 820.437 | 356.7256 | 266.7841 | 1110.7161 | 3873.7751 | 3674.072 | 3063.2175 | 6171.3492 | 894.1886 | 1209.6267 | 403.3846 | 44.1862 | 41.9282 | 767.8502 | 0.01777433 | 0.09537497 | 0.012754892 | 0.702215026 |
